# On the use of ^31^P NMR for the quantification of hydrosoluble phosphorus-containing compounds in coral host tissues and cultured zooxanthellae

**DOI:** 10.1038/srep21760

**Published:** 2016-02-23

**Authors:** Claire Godinot, Marc Gaysinski, Olivier P. Thomas, Christine Ferrier-Pagès, Renaud Grover

**Affiliations:** 1 Centre Scientifique de Monaco, CSM, 8 Quai Antoine 1er, 98000 Monaco, Principality of Monaco; 2 LEA CSM-CNRS 647 ‘Biosensib’, Monaco, Principality of Monaco; 3 University Nice Sophia Antipolis, Plate-Forme Technologique de Chimie, Faculté des Sciences, Parc Valrose, 06108 Nice, France; 4 University Nice Sophia Antipolis, Institut de Chimie de Nice - EEIC, UMR 7272 CNRS, Parc Valrose, 06108 Nice, France; 5 Institut Méditerranéen de Biodiversité et d’Ecologie Marine et Continentale, UMR 7263 CNRS, IRD, Aix Marseille Université, Avignon Université, Station Marine d’Endoume, Rue Batterie des Lions, 13007 Marseille, France

## Abstract

^31^P Nuclear Magnetic Resonance (NMR) was assessed to investigate the phosphorus-containing compounds present in the tissues of the scleractinian coral *Stylophora pistillata* as well as of cultured zooxanthellae (CZ). Results showed that phosphorus-containing compounds observed in CZ were mainly phosphate and phosphate esters. Phosphate accounted for 19 ± 2% of the total phosphorus compounds observed in CZ maintained under low P-levels (0.02 μM). Adding 5 mM of dissolved inorganic phosphorus (KH_2_PO_4_) to the CZ culture medium led to a 3.1-fold increase in intracellular phosphate, while adding 5 mM of dissolved organic phosphorus led to a reduction in the concentration of phosphorus compounds, including a 2.5-fold intracellular phosphate decrease. In sharp contrast to zooxanthellae, the host mainly contained phosphonates, and to a lesser extent, phosphate esters and phosphate. Two-months of host starvation decreased the phosphate content by 2.4 fold, while bleaching of fed corals did not modify this content. Based on ^31^P NMR analyses, this study highlights the importance of phosphonates in the composition of coral host tissues, and illustrates the impact of phosphorus availability on the phosphorus composition of host tissues and CZ, both through feeding of the host and inorganic phosphorus enrichment of the CZ.

Scleractinian corals form reefs in shallow, oligotrophic waters, where nutrient, and phosphorus concentrations in particular, are relatively low compared to other aquatic systems^1^,^2^,^3^. Nevertheless, phosphorus enters into the composition of many biological molecules (deoxyribonucleic and ribonucleic acids, adenosine triphosphate, phospholipids …) and plays a central role in life processes, often limiting coral calcification and photosynthesis^4^,^5^,^6^. Once taken up, phosphorus is however efficiently retained by corals and their symbiotic dinoflagellates, called zooxanthellae, which are harbored within coral endodermal cells (Fig. 1). This is one of the reasons why corals can flourish in nutrient-poor waters. Due to the physiological importance of phosphorus-containing compounds, associated with their scarcity in reef waters, the knowledge of phosphorus cycling pathways and speciation within corals is of key importance for a better understanding of reef ecosystems. Both are still relatively poorly understood in corals, while they have been extensively studied in other aquatic and marine organisms. Phosphorus-containing molecules, such as phosphate ions, polyphosphates, phosphate esters (molecules with O-P or C-O-P bonds) and phosphonates (molecules with a C-P bond), have been described and/or quantified in cnidarians and other marine invertebrates^7^,^8^,^9^. Phosphate is a source of phosphorus directly available for the cellular metabolism, and its internal concentration in algae is linked with the state of algal differentiation^10^, and with the external phosphate concentrations^11^,^12^,^13^. Polyphosphates are considered as phosphorus storage molecules and cation sequestration complexes in living cells^14^, but they are not systematically present in marine organism such as algae or giant clams, in which two studies failed to observe them^15^,^16^. The recent discovery of polyphosphate granules produced by bacteria in sponges underlines the importance of the phosphate cycle in the context of the “Darwin’s paradox”: paradox”: How can high production flourish in low-nutrient conditions^17^? Finally, phosphonates which commonly occur as lipid, proteinaceous and glycoprotein conjugates^18^,^19^,^20^, may represent a major source of phosphorus for marine microorganisms^21^,^22^,^23^,^24^, and cnidarians^18^,^25^,^26^,^27^, accounting for 10% to as much as 50% of cellular particulate phosphorus. Although their metabolic importance remains unclear^9^,^20^, the presence of resistant C-P bonds in lipids has been suggested to provide increased strength and protection to organisms that lack protective outer coatings of chitin or cellulose^28^. All the above phosphorus-containing molecules occur at all levels of corals and zooxanthellae metabolism, and especially in membrane composition and lipid reserves. They therefore play a key role in the energetics and functioning of the symbiosis. A better knowledge of their composition and concentration in both members of the symbiosis would strengthen our knowledge on P-cycling in corals and on the importance of phosphorus and phosphorus-containing molecules for the growth and maintenance of corals.

In various cnidarians and in symbiotic clams, phosphorus-containing molecules have also been studied^16^,^18^,^27^,^29^,^30^,^31^, and some have been quantified. For example, ATP content in coral tissues has been estimated to be 8–53 μg ATP g^−1^, with a decrease with coral bleaching^30^ or with starvation in the symbiotic anemone *Aiptasia pulchella*^27^. Phospholipids have been estimated to represent ca. 25% of anemone lipids, half of which are present in the symbionts^29^. In various sea anemones, it was reported that a specific phosphonate, aminoethyl phosphonate (AEP), is often abundantly present^18^,^25^,^26^,^27^. So far most studies on cnidarians have focused on only one kind of phosphorus-containing compounds, and attempts at quantification have been rare. The simultaneous detection of all types of hydrosoluble phosphorus-containing molecules can be achieved by the use of nuclear magnetic resonance (NMR) spectroscopy in solution^32^. ^31^P NMR spectroscopy typically distinguishes phosphate, polyphosphates, phosphonates, and various phosphates esters by their chemical shifts (Fig. 2). Despite the key information that can be gained using ^31^P NMR spectroscopy on coral samples, there are only few previous studies in the literature that reported the use of this technique on cnidarians^26^,^27^,^31^. Two of them used *in vivo*^31^P NMR on live polyps of the sea anemones *Anemonia pulchella* and *A. viridis* to investigate host adenylate ratio (ATP/(ADP + ATP)), but failed to observe any phosphorus signal when trying to examine freshly isolated zooxanthellae^27^,^31^. The last study examined the phosphate and phosphonates composition of extracts from the sea anemone *Bunadosoma* sp., but more with the aim of refining existing hydrolytic procedures of phosphonates in marine samples rather than examining the phosphorus metabolism^26^.

The aim of the present study is to assess the use of ^31^P NMR spectroscopy for the determination and quantification of the hydrosoluble phosphorus-containing compounds present in the two components of the coral symbiosis, in order to pave the way for an understanding of the phosphorus metabolism in corals. For this purpose, the robustness of the ^31^P NMR spectroscopy tool was first examined in the host and zooxanthellae of the scleractinian coral *Stylophora pistillata*. The potential of this tool was then examined with a series of experiments, which aimed at determining the impact of several environmental conditions on the phosphorus composition of cultured zooxanthellae (CZ) and coral host tissues. We targeted environmental conditions which are known to have a major impact on the phosphorus composition of corals from the literature. For CZ, we assessed the impact of phosphorus enrichment and culture age, considering that different growth stages are known to affect the phosphorus composition in other algae^10^. Host feeding constitutes an organic phosphorus source, which is known to affect coral uptake of dissolved phosphorus^6^,^33^,^34^, and may therefore modify the content of different phosphorus derivatives within the symbiosis. We thus examined the impact of feeding on host tissue phosphorus composition. Finally, we assessed the impact of bleaching on the phosphorus composition of host tissues, as symbiont loss may affect the phosphorus provisions of the host. This study thus broadens the array of tools enabling the assessment of coral nutrient metabolism, with the potential to determine the impact of various environmental stressors on the phosphorus metabolism of corals.

## Results

### Cultured zooxanthellae

**Figure 3:**
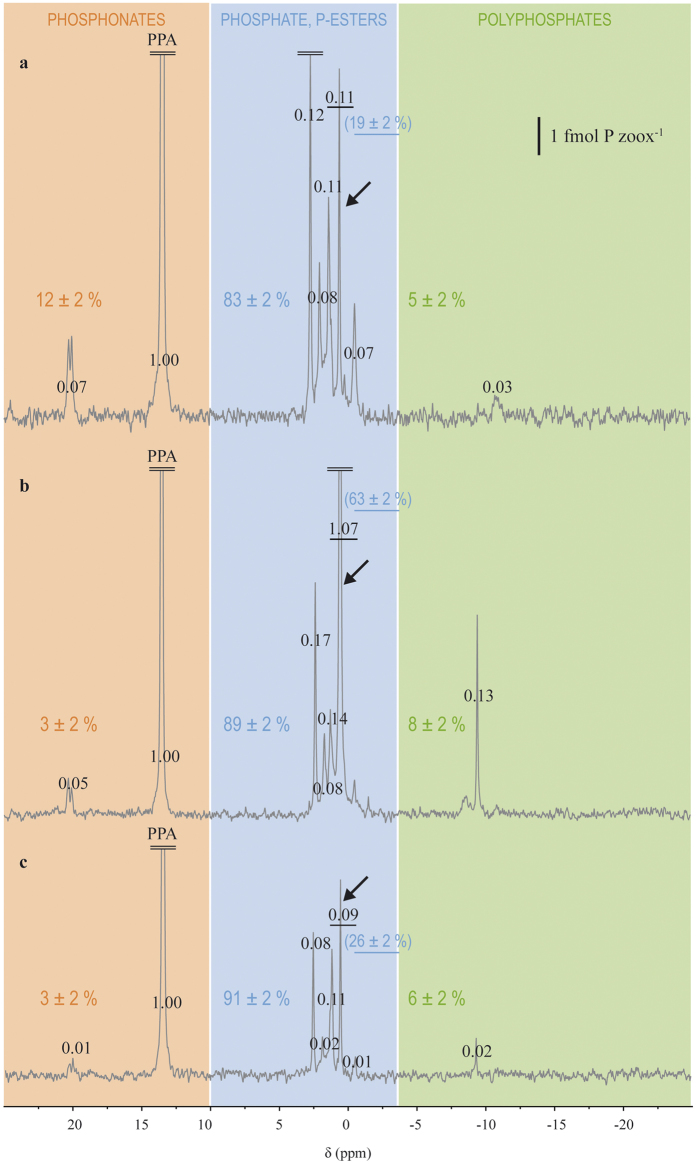
Impact of the phosphorus enrichment in the culture medium (ASP-8A medium, which contains 0.02 μM phosphate) on the phosphorus composition of cultured zooxanthellae (CZ). (**a**) control CZ enriched with ASP-8A medium. (**b**) CZ enriched with 5 mM phosphate (DIP). (**c**) CZ enriched with 5mM of beta-glycerophosphate (DOP). The three spectra were vertically scaled to the scale given in the upper-right corner. Values in black on the spectra represent the integration (relative to a PPA integration of 1.00 for 5 μmol of PPA). Colored values indicate the relative content of each class of compounds indicated by the shaded areas, and the underlined ones in brackets indicate the relative phosphate content. The underlined value and the arrow indicate the phosphate peak.

The phosphorus compounds observed in CZ were mainly phosphate and phosphate mono- and diesters, which accounted for 83–91% of the total phosphorus compounds, and, to a much lesser extent, polyphosphates (5–8%) and phosphonates (3–12%; Fig. 3). Phosphate accounted for 19 ± 2% of the total phosphorus compounds observed in the unenriched control zooxanthellae (Fig. 3a). Overall, dissolved inorganic phosphorus (DIP) enrichment led to an increase in the contents of phosphate, phosphate esters and polyphosphates (Fig. 3 and Table 1). In contrast, dissolved organic phosphorus (DOP) enrichment led to a reduction in the content of all phosphorus compounds observed on the spectra. For example, a 3.1-fold increase in phosphate content was observed in the presence of DIP, while a 2.5-fold decrease was obtained with DOP enrichment (Table 1).

Ageing of CZ cultures impacted the phosphate content of CZ, which increased by 2.6-fold between day 15 and 38 (Table 1). All other peaks increased as well, but the number of compounds present was not modified (data not shown).

### Host tissues

**Figure 4:**
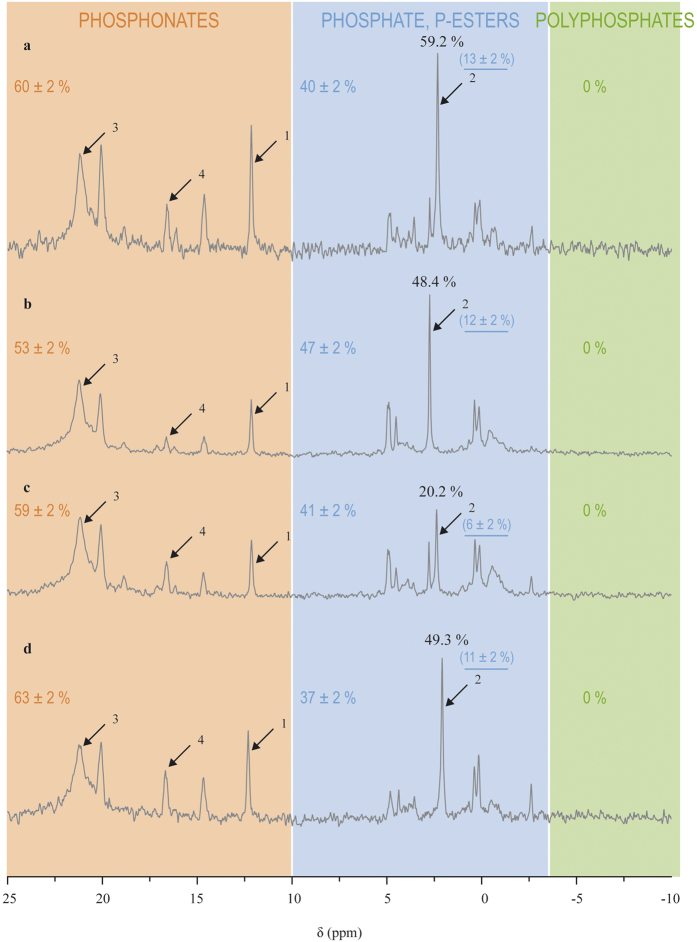
Impact of host feeding (**a–c**) and symbiotic status (**b,d**) on the phosphorus composition of host tissues. (**a**) highly fed corals. (**b**) control corals. (**c**) starved corals. (**d**) bleached corals. Arrow 1 indicates the PPA standard (0.5 μmol), arrow 2 the phosphate peak, arrow 3 the 21.2 ppm phosphonate peak, and arrow 4 the peak tentatively identified as an AEP derivative. Percentages in black indicate the proportion of phosphate relative to the 21.2 phosphonate peak. Colored values indicate the relative content of each class of compounds indicated by the shaded areas, and the underlined ones in brackets indicate the relative phosphate content.

In contrast to cultured zooxanthellae, the phosphorus compounds observed in host tissues were mainly a large diversity of phosphonates, which accounted for 53–63% of the total phosphorus compounds observed, and to a lesser extent, phosphate and phosphate esters, which accounted for 37–47% (Fig. 4). Phosphate accounted for 6–13% of the total phosphorus compounds observed.

Important host feeding increased the ratio of phosphate relative to the phosphonate peak at 21.2 ppm compared to that of control corals, while host starvation induced a decrease of this ratio (Table 2 and Fig. 4). Indeed, control corals had 48.7 ± 0.3% phosphate (relative to the phosphonate peak at 21.2 ± 0.01 ppm), a percentage which increased to 61.2 ± 1.2% with a high feeding regime, and which decreased to 20.2% with a 2-months starvation. Symbiont loss (i.e., bleaching) did not modify the phosphate content (49.3%) as compared to control corals. No other major variations occurred in the rest of the NMR spectra (Fig. 4).

**Figure 5:**
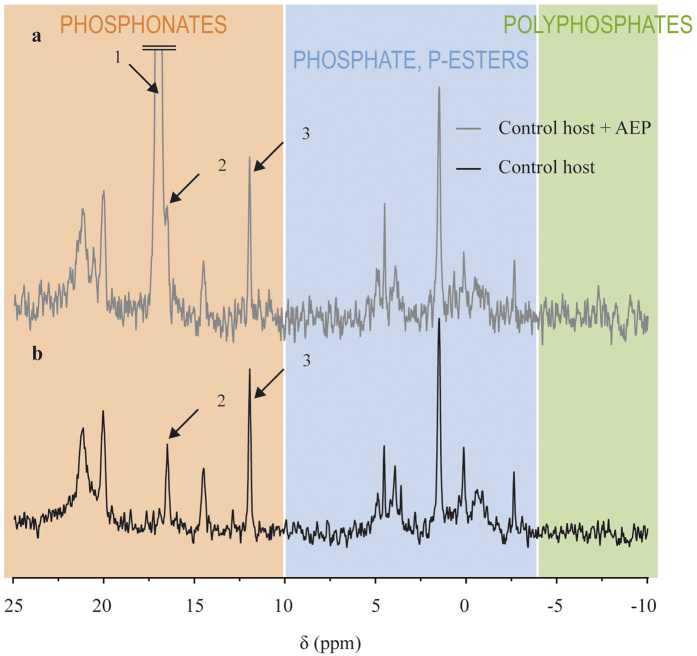
Spiking of the bleached host sample with 5.0 μmol of AEP (**a**) and comparison with the pure bleached host sample (**b**). Arrow 1: AEP peak, arrow 2: phosphonate peak closest to the AEP peak, and arrow 3: 0.5 μmol PPA.

Spiking the bleached host sample with 5.0 μmol of AEP (Fig. 5) showed that peak 4 on Fig. 4 is not AEP, since the shift of that peak (16.55 ppm, peak 2 on Fig. 5) did not match the shift of the AEP peak (17.1 ppm, peak 1 on Fig. 5).

### Freshly isolated zooxanthellae (FIZ)

As detailed in the ESM, results from a preliminary experiment (cross-contamination) showed that the centrifugation technique used to isolate FIZ from host cells led to a contamination of FIZ spectra by host tissues, while the reverse was not observed. Therefore, we conclude in the ESM that the separation technique by centrifugation was not suitable for FIZ observations by ^31^P NMR, and we then decided to discard the subsequent FIZ spectra, while keeping the results obtained for the host.

## Discussion

All hydrosoluble phosphorus compounds will be detected within a sample by NMR spectroscopy in solution, while phosphorus nuclei involved in insoluble or immobilized compounds are usually not seen due to heterogeneity factors, as it is usually the case for membrane phospholipids for example, which generally form micelles in water solutions. Nucleic acids, especially those involved in large macromolecules such as DNA and RNA, also fall in that category, and were not observed in this study. We therefore focused on hydrosoluble species of phosphorus in this “in solution” NMR study that are of high interest as available substances for different kind of metabolisms.

The developed NMR protocol was not applicable to FIZ. A previous study on the anemone *A. pulchella* also failed to develop a protocol for FIZ examination, and only shows background noise for FIZ spectra^27^. However, in that latter study, failure may have been due to a lack of material, since Steen *et al.* performed the analyses with only 2.3 × 10^6^ FIZ per sample (0.9 × 10^6^ FIZ mg animal prot^−1^, and a protein content of 1.0 mg animal protein per anemone), which is insufficient for the sensitivity of ^31^P NMR. In the present study, FIZ concentrations were one or two orders of magnitude higher (0.5–3.0 × 10^8^ FIZ per sample), and the lack of applicability of the ^31^P NMR protocol was not due to a low compound concentration, but rather to contamination by host tissues, and to losses of soluble phosphate from the zooxanthellae pellet, during separation by centrifugation (See Supporting Information).

Conversely, symbiont contamination of the host tissue was negligible. Centrifugation has been used in countless of physiological studies to separate zooxanthellae from host tissues, but its efficiency has been less frequently examined. In the coral *Pocillopora damicornis*, contamination of FIZ by host tissues was of ca. 6.0%, and that host tissue contamination is of ca. 1.3%, therefore estimating that the separation of algae and animal tissue by centrifugation is about 95% efficient and that mutual contamination of fractions does not constitute a significant error^35^. Other studies on green hydra estimated similar separation efficiencies of ca. 90%^36^,^37^, but highlighted that contamination by the remaining 10% may be very high for some applications^37^. Alternative separation techniques such as centrifugation through a Percoll gradient or filtration have been successively used to reduce contamination of FIZ by host tissues^38^,^39^,^40^, but their rather low yield due to material losses may hamper their applicability to NMR. Additional studies are thus required to achieve contamination-free isolation of FIZ from host tissues before ^31^P NMR can be successfully applied to symbiotic zooxanthellae; and while experiments performed with CZ are informative, one should keep in mind that they do not give a full account of the phosphorus metabolism and composition of the holobiont.

Host tissues and cultured zooxanthellae differed in their phosphorus composition, with abundant and diverse phosphonates in the host (four peaks) versus scarce phosphonates in CZ (one peak), and polyphosphates only observed in CZ. Additionally, P-ester regions covered a wider spectral range in host tissues than in CZ, and are therefore presumably representative of different P-esters chemical diversities. A previous ^31^P NMR study on *A. pallida* revealed four peaks in the polyphosphate region for ATP, ADP, adenylate and reductants^27^. Similar spectra were observed in *Anemonia viridis*^31^. Polyphosphates were absent for host tissues in the present study, and only one peak was observed in the polyphosphate region for CZ, which is consistent with results from previous studies on algae^15^, and giant clams^16^. On the light of the results obtained in sponges^17^, polyphosphates may be present in granule forms as in this case they would not be observed by NMR in solution. However, to date there is no evidence of the presence of such granules in corals and this phenomenon may be restricted to sponges and their associated bacteria. It is important to underline that some polyphosphates may have been present in the host and CZ, but at concentrations too low to afford observable signals by NMR.

The relative absence of phosphonates in CZ differs from previous reports that phosphonates can occur in a large variety of marine protozoans, including dinoflagellates^7^,^8^. Conversely, the diversity and abundance of phosphonates in host tissues are in agreement with previous reports which showed that they may represent as much as 30–50% of the total phosphorus content in cnidarians^18^,^26^,^27^. While in sea anemones, it was reported that AEP is often abundantly present^18^,^25^,^26^,^27^, in the present study, AEP was not found in the host. The peak observed in the vicinity of the AEP peak (peak 1 on Fig. 5) may have been one of its common derivatives, methylaminoethyl phosphonate, or amino phosphonopropanoate, that is also frequently observed in anemones^9^,^25^,^27^. In *A. pallida*, it was hypothesized that phosphonates were membranar phosphonolipids, because of the typical asymmetric shape of the corresponding peaks on the NMR spectra, which is indicative of membrane components in NMR^27^. The same conclusion may also be given in the present study for the phosphonates evidenced in the host spectra (Fig. 4).

In CZ, the phosphate content measured in the present study (between 1.3 and 10.3 fmol P zooxanthellae^−1^; Table 1), and the range of calculated phosphate concentrations (between 3–7 and 26–54 mmol P L^−1^; Table 1) are, to the best of our knowledge, the first report of phosphate concentrations in zooxanthellae cells. The phosphate content is consistent with reports of total phosphorus content of zooxanthellae from *S. pistillata* (between 40 and 60 fmol P zooxanthellae^−1^)^34^,^41^, but is two to three orders of magnitude lower than reports of total phosphorus content of zooxanthellae from the coral *P. damicornis* (5–10 pmol P zooxanthellae^−1^)^33^. The millimolar concentration calculated in the present study is consistent with concentrations found in other algal cells (between 1 and 15 mmol P L^−1^)^42^,^43^,^44^,^45^.

The ^31^P NMR protocol presented here allowed for the examination of the impact of environmental conditions on the phosphorus composition of CZ and host tissues.

The intracellular phosphorus enrichment of CZ with ageing, and especially their phosphate enrichment, is consistent with previous reports that intracellular phosphate pools increase with the phase of differentiation (i.e. culture age) of algal cultures^10^.

DIP enrichment of the CZ cultures led to an intracellular phosphorus enrichment, with a 3.1-fold increase in phosphate content and phosphate concentration, from 12.4 to 38.3 mmol P L^−1^. Previous NMR studies had similarly shown that phosphate enrichment, and phosphate depletion, of algal cultures, respectively led to an increase, and a decrease, of the intracellular phosphate content^11^,^12^,^13^. Conversely, DOP enrichment led to a 2.5 fold decrease of the phosphate content and concentration of CZ. One hypothesis to explain such poor use of DOP by CZ in the DOP-enriched cultures is that they had a low alkaline phosphatase activity (APA), as already observed in some cultures^6^, and therefore could not optimally use DOP.

In the host, intracellular phosphate increased with feeding, which is in agreement with a recent report that zooplankton feeding provides DIP and DOP to corals (ca. 3 μg cm^−2^ d^−1^ for DIP + DOP)^46^. The lack of impact of bleaching on the phosphate content was probably linked to that strong impact of feeding, as bleached corals were fed by the same nutritive regime as control corals. Furthermore, as zooxanthellae photosynthates transferred to the host mostly consist in carbon- and nitrogen-rich compounds such as glycerol and maltose, but not in phosphorus-rich compounds, no major impact of symbiont loss on host phosphorus composition is expected^47^,^48^,^49^,^50^.

The importance of phosphonates in the composition of host compounds (53–63% of total host phosphorus compounds) was highlighted for the first time, and the response of coral and CZ phosphorus composition to ambient phosphorus pools was established. Since phosphonates were only abundant and diverse in the host, their synthesis is likely not dependent upon the symbionts. Also, symbiont loss did not impact the phosphorus composition of the host, demonstrating that, as far as phosphorus composition is concerned, the phosphorus metabolism of the host is not greatly impacted by the symbionts. Therefore, it seems important to follow this study by attempts at 1) identifying the phosphonates present in the host in order to elucidate their biological role, and at 2) improving the isolation of FIZ for ^31^P NMR measurements, in order to examine the phosphorus composition of the symbionts as well and to examine the dependency of their phosphorus composition upon host metabolism. Solid-state NMR analysis would also afford valuable information on the composition and distribution of phosphorus compounds within the organisms.

## Methods

### Experiments with cultured zooxanthellae

Zooxanthellae from the species *Symbiodinium microadriaticum*, initially isolated from the scleractinian coral *Stylophora pistillata*, were cultured in 125-ml Erlenmeyer flasks containing autoclaved ASP-8A medium at pH 8.2 and maintained in an incubator (Sanyo, UK). The ASP-8A medium contained 0.02 μM of phosphate, ammonium, and nitrate. Growth temperature was 25.0 ± 0.5 °C, and light was provided by daylight fluorescent tubes (15,000 K) at 80 μmol photons m^−2^ s^−1^ on a 14 h:10 h light:dark cycle.

In the first experiment, the impact of dissolved phosphorus enrichment was tested on the CZ phosphorus composition. Logarithmically growing CZ (aged 19 days^51^) were enriched with either: i) 1 mL of ASP-8A medium (control), ii) 1 mL of a stock solution of KH_2_PO_4_ (for a final concentration of 5 mM inorganic phosphate), iii) 1 mL of a stock solution of beta-glycerophosphate (for a final concentration of 5 mM organic phosphorus). The CZ from the 3 treatments were collected 3 days after the enrichments for ^31^P NMR measurements, and were thus 22-days old at the time of NMR measurements.

In a second experiment, we tested whether the phosphorus composition changed with the growth phase. Logarithmically growing CZ (aged 15 days) were therefore compared to stationary-phase CZ (aged 38 days)^51^.

All CZ used for the NMR measurements were collected at the same time of day (between 09:00 and 10:00 hrs), and were washed 3 times (centrifugation at 2000 *g*, for 5 min, at 4 °C) in a buffer (called the “cytoplasmic buffer”)^52^. This buffer contained approximate cytosolic concentrations of major ions in cnidarian cells^53^,^54^. Phosphatase inhibitors were added to prevent the hydrolysis of organic phosphorus compounds (50 mM NaF, 1 mM Na_3_VO_4_) and mannitol to adjust osmolarity to 1,100 mOsm l^−1 ^55. pH of the “cytoplasmic buffer” was adjusted to 7.4 with 1 M HCl and with 25 mM Pipes. For each NMR measurement, a 1 mL aliquot of the CZ was taken for cells counts (a yield of 2.2–5.2 10^8^ zooxanthellae was typically harvested). After a final centrifugation (2,000 *g*, for 5 min, at 4 °C), 6 mL of boiling 50 mM EDTA was added to the CZ pellet and the homogenate was boiled for 7 min at 100 °C^16^. The mixture was then centrifuged at 12,000 *g* for 10 min, the pellet discarded, and the supernatant freeze-dried.

### Experiments with cultured corals

Colonies of the scleractinian coral *S*. *pistillata* were maintained in aquaria supplied with natural seawater (exchange rate 2% h^−1^) containing low phosphate (<0.05 μmol L^−1^), nitrate (<0.4 μmol L^−1^) and ammonium (<0.5 μmol L^−1^) concentrations. Seawater salinity was 38.2, pH was 8.1 ± 0.1, temperature was maintained at 25.0 ± 0.5 °C, and irradiance was 150 μmol photons m^−2^ s^−1^.

We first tested the impact of feeding on the phosphorus composition in coral hosts. Corals were subjected to three different feeding regimes during two months: i) “highly fed” corals, fed 5 times per week with both frozen krill and live *Artemia salina* nauplii, ii) “control” corals, fed 2 times per week with the same food, and iii) “starved” corals, which were not fed for 2 months before the NMR measurements.

In the following experiment, we tested the impact of bleaching (symbiont loss) on the phosphorus composition of the coral host, by comparing symbiotic and highly bleached (aposymbiotic) *S. pistillata* hosts. Bleached colonies were prepared by culturing colonies for 6 weeks in the dark, and fed a regime similar to the control corals from the previous experiment. Microscopy observations confirmed that symbiont density was 3 orders of magnitude lower in bleached (6.7 × 10^3^ zooxanthellae cm^−2^) than in normal colonies (2.5 × 10^6^ zooxanthellae cm^−2^).

All corals used for the NMR measurements were collected at the same time of day (between 09:00 and 10:00 hrs). The isolation of coral tissues from the skeleton and the subsequent separation of host tissues from FIZ were performed on ice (0–4 °C), in the “cytoplasmic buffer” described above. For each NMR measurement, 20 branch tips (5.0 ± 1.0 cm long, 1.0 ± 0.2 cm wide) of *S. pistillata* colonies were sampled using surgical bone cutters, and crushed with a hammer. The skeletal fragments were covered with the “cytoplasmic buffer”, and the tissues were removed by agitation in a flask^35^. The crude suspension was then filtered by gravity through a 40 μm mesh net to break cell aggregates, homogenized in a Dounce homogenizer, and sonicated for 10 minutes to break the animal cells and separate them from the zooxanthellae (0–4 °C). Separation was completed by 3 successive centrifugations at 2000 *g*, for 5 min, at 4 °C. The animal supernatants were pooled, and 5 μmol of phenylphosphonic acid (PPA) were added to act as an internal calibration standard during NMR measurements. Samples were then lyophilized during 48 h.

### ^31^P NMR measurements

Just before the NMR experiments, the freeze-dried samples were redissolved in 4 mL buffer. This buffer (hereafter referred to as the “NMR buffer”) contained 50 mM TES (Sigma Aldrich, CAS Number 7365-44-8), 10% D_2_O in deionized water (DIW), and had a pH of 7.4^12^,^56^. For all measurements, a quantification of the peaks was achieved by adding a known quantity (between 0.5 and 5.0 μmol) of phenylphosphonic acid as an internal quantification standard before freeze-drying.

All ^31^P NMR spectra were recorded at 298 ± 0.1 K (temperature control BCU 05, BVT 3000), on a BRUKER AVANCE DRX 500 spectrometer (500.13 MHz proton frequency and 202.45 MHz ^31^P frequency) equipped with a direct probehead (10 mm PH BBO H-D). D_2_O was used for internal lock purposes. H_3_PO_4_ was used as an external reference for spectrum calibration to express the chemical shift (δ, expressed in parts per million, ppm) of phosphorus compounds. All NMR experiments were carried out using pulse sequences supplied by the spectrometer manufacturer (BRUKER – TOPSPIN 2.1). Each ^31^P NMR spectrum was acquired using 20.3 kHz Spectral Width, 64 K complex data point, an acquisition time of 1.6 s, a relaxation delay of 1.2 s, a number of scan between 19000 and 20000, a number of dummy scan of 32, and a 45° flip angle pulse width. ^31^P NMR experiments where performed without ^1^H decoupling. The 45° pulse was set at 6.50 μs. Prior to Fourier transformation, free induction decays (FIDs) were multiplied by an exponential line broadening function of 10 Hz. The resulting spectra were manually phased and baseline corrected.

^*31*^*P NMR calibration.*  Preliminary tests were performed to ensure that NMR experiments were run under optimal conditions with coral samples (See Supporting Information). We i) showed that PPA is a good internal calibration standard for ^31^P NMR experiments involving corals, and ii) determined an equation to calculate the phosphate chemical shift comparing with PPA (which greatly helped to identify the phosphate peak in the generally dense region of the spectra where it occurs). We also iii) performed a cross-contamination experiment which demonstrated that the centrifugation technique used to separate FIZ and host tissue is not suitable for the observation of zooxanthellae freshly isolated from coral tissues by ^31^P NMR. Therefore, only host tissues were subsequently examined.

Duplicated experiments showed that measures were highly repeatable (Table 1), and that the content of each hydrosoluble phosphorus compound could be determined with a precision of ±2%, and the calibration curve performed between pH and the *δ*_P_ was useful to examine the phosphate content of all samples (See Supporting Information).

*Phosphorus quantification.*  Phosphate content was quantified for each CZ and host sample using the ratio of phosphate to PPA peaks integration (values are expressed as μmol of P). For CZ, results were normalized both to cell concentration and cell volume (expressed in fmol of phosphate per zooxanthellae, as well as in mmol phosphate L^−1^). This latter parameter was calculated using a mean diameter of 7–9 μm for CZ, which gives a total volume of 1.8–3.8 10^−13^ L zooxanthellae^−1^, considering zooxanthellae as spherical. The so-called phosphorus concentration obtained by this calculation gives the range of phosphorus concentration in the cytoplasm of the CZ, but one should note that the subdivision of zooxanthellae cells by organelles was not taken into account in this rough calculation. For animal host tissues, on the contrary, normalization by cell numbers was not possible (no counting possible), and phosphate content was quantified using the same method as described by Steen^27^. Following this approach, the signal assigned to a specific phosphonate (*δ* 21.2 ± 0.01 ppm) that was always present on the spectra, and was assumed constant (*i.e.*, concentration independent of environmental conditions), and the phosphate content was calculated relative to that phosphonate peak (values are expressed as percentages). For CZ and host tissues, phosphate, phosphate esters, phosphonate and polyphosphate contents were also calculated relative to the total phosphorus content observed on each spectra (values expressed as percentages).

## Additional Information

**How to cite this article**: Godinot, C. *et al.* On the use of ^31^P NMR for the quantification of hydrosoluble phosphorus-containing compounds in coral host tissues and cultured zooxanthellae. *Sci. Rep.* **6**, 21760; doi: 10.1038/srep21760 (2016).

## Supplementary Material

Supplementary Information

## Figures and Tables

**Figure 1:**
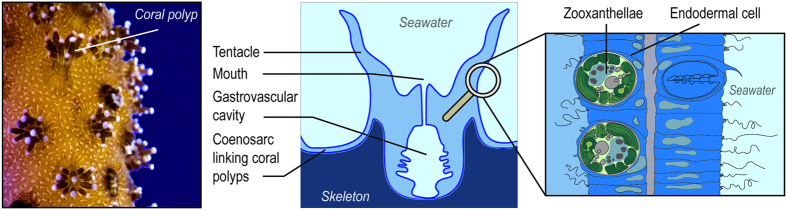
Location of zooxanthellae within the coral host (Pictures by C. Godinot).

**Figure 2:**
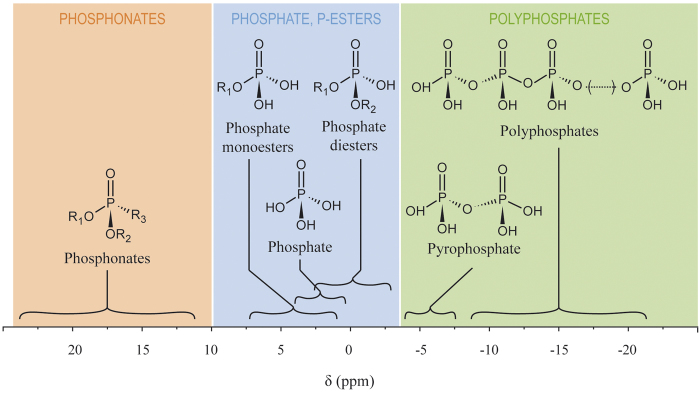
Chemical shifts (δ) of biological phosphorus compound classes expected to occur in coral cells at physiological pH. Chemical shifts are a synthesis of published values from the literature and from values obtained during this study. Shaded areas indicate the three general classes of phosphorus compounds considered in this study: phosphonates, phosphate and phosphate-esters, and polyphosphates.

**Table 1:** Variation of phosphate relative to the phosphonate peak with a δ of 21.2 ppm in host tissues from corals submitted to three different feeding regimes and from bleached corals.

Treatment	Phosphate content		Phosphonate content		Percentage of phosphate relative to phosphonate
	(μmol P.sample^−1^)	(μmol P.g^−1^ sample)	(μmol P.sample^−1^)	(μmol P.g^−1^ sample)	(%)
Highly fed (larger sample)	21.0	2.8	33.2	4.2	63.3
Highly fed (smaller sample)	7.9	1.0	13.4	1.6	59.2
Control (larger sample)	41.0	5.1	83.7	10.4	49.0
Control (smaller sample)	13.6	1.6	28.1	3.3	48.4
Starved	7.5	0.9	37.1	4.5	20.2
Bleached	8.8	1.2	17.8	2.6	49.3

The first two treatments (highly fed and control) were duplicated, each time with a c.a. 3 fold difference in coral biomass used for the extraction, in order to verify that sample biomass did not influence the results in the range tested. Data are normalized per sample and per sample weight.

**Table 2:** Variation of CZ phosphate content at various growth stages and with various sources of phosphorus in the culture medium (ASP-8A medium, which contains 0.02 μM phosphate).

Treatment	Zooxanthellae content	Phosphate content	Phosphate content of CZ	Phosphate concentration in CZ
	(10^8^ zooxanthellae.sample^−1^)	(μmol P.sample^−1^)	(fmol P zooxanthellae^−1^)	(mmol P L^−1^)
15-days old CZ	2.19	0.55	2.51	9.37
22-days old CZ	3.05	1.02	3.34	12.4
38-days old CZ	4.07	2.70	6.63	24.75
22-days old CZ + DIP	5.21	5.35	10.3	38.30
22-days old CZ + DOP	3.36	0.45	1.34	5.0

15 and 22-days old CZ are logarithmically growing, while 38 days old-CZ are in stationary phase^51^. The culture medium was enriched with either ASP-8A medium (22-days old CZ, control), 5 mM of KH_2_PO_4_ (22-days old CZ + DIP), or 5 mM of beta-glycerophosphate (2-days old CZ + DOP).
