# Supplementary material for: On the use of 31P NMR for the quantification of hydrosoluble phosphorus-containing compounds in coral host tissues and cultured zooxanthellae
Source: Sci Rep. 2016 Feb 23;6:21760. doi: 10.1038/srep21760 (PMC4763230; doi:10.1038/srep21760)
Supplement: Supplementary Information [file srep21760-s1.pdf]

Supporting information for

On the use of ^{31}P-NMR for the quantification of hydrosoluble phosphorus-containing compounds in coral host tissues and cultured zooxanthellae

Claire Godinot[1,2], Marc Gaysinski[3], Olivier P. Thomas[4,5,], Christine Ferrier-Pagès[1,2] & Renaud Grover[1,2,]**

^{31}P-NMR calibration

Preliminary tests were performed to ensure that NMR experiments were run under optimal conditions with coral samples.

We first measured the chemical shift of two solutions containing in total six identified phosphorus-containing molecules. These molecules were chosen because they cover the range of chemical shifts for molecules expected to occur in coral samples. The molecules and their chemical shifts are presented on Figure 6. The first solution was made of 5.0 µmol of both PPA and aminoethylphosphonic acid (AEP) in the “NMR buffer". The second solution was made of 5.0 µmol of PPA, of KH_2PO_4, of beta-glycerophosphate, of adenosine triphosphate (ATP), and of polyphosphates in the “NMR buffer". Polyphosphates were used in the form of sodium hexametaphosphate 70 FG (thermPhos, Germany), which has a specified average chain length of 30. Observed resonances were compared to published values from the literature. The chemical shifts of the six authenticated compounds used for the first preliminary test are presented in Figure 6. This preliminary test confirmed that no overlapping occurs using PPA as a standard. Spectra obtained with coral cells and CZ also confirmed the absence of overlapping, making PPA a good internal calibration standard for ^{31}P-NMR experiments involving corals.

We then determined the influence of pH on the NMR observations, since it was observed that pH may slightly vary between experiments despite the use of the "NMR buffer" (Figure 7).

The second preliminary test therefore consisted in the determination of the chemical shift of both P atoms from phosphate ions (δ PO_4) and P atom from PPA (δ PPA) as a function of pH using various buffers containing 5 mM KH_2PO_4 and 5 mM PPA in 50 mM TES, 10% D_2O, with pH values of 4.25, 5.0, 5.5, 6.0, 6.5, 7.0, 7.5, and 8.0. Chemical shifts were referenced to 85% phosphoric acid. In order to be able to calculate the δ PO_4 from the observed δ PPA at any pH comprised in the 4.5 - 8.0 range, we empirically calculated the mathematical equation of the titration curves of phosphate and PPA with the GraphPad Prism software (version 5.0; San Diego, CA, USA), using logarithmic regressions between

pH and δ PPA and between pH and δ PO_4. Results from the second preliminary test are presented on Figure 7. The following equation (Eqn. 1) was obtained to calculate the phosphate shift (δ PO_4) relative to the PPA shift (δ PPA):

$$\delta\, PO_4 = a + \frac{b + c \times \delta\, PPA}{d + e \times \delta\, PPA} \qquad Eqn.1$$

with a = 2.83, b = 2.97 10^8, c = - 2.56 10^7, d = 8.24 10^7, and e = - 4.59 10^6. This equation greatly helped to identify the phosphate peak in the generally dense region of the spectra where it occurs, and was used for each experiment we performed with corals or zooxanthellae.

In some of the coral samples, the phosphate peak was further confirmed by spiking the samples with 5.0 µmol of KH_2PO_4 after the first spectra acquisition. Spiking of one of the host samples with 5.0 µmol of AEP was also performed to confirm the putative identification of this compound in coral spectra, as AEP has been reported to be often abundantly present in other cnidarians (Kittredge et al. 1962; Quin 1964; Henderson et al. 1972; Steen 1986).

Finally, we tested the efficiency of the centrifugation technique used to isolate host tissues from zooxanthellae cells. We tested the protocol described in the article using different mesh sizes (10-40 µm), sonication durations (3-15 min), as well as centrifugation speeds (500-5000 g) and durations (1-15 min). The parameters reported in the materials and methods of the article are the ones that gave the best results in terms of isolation (purity of the animal supernatant and integrity of the zooxanthellae in the pellet) and still afforded a sufficient zooxanthellae count at the end of the procedure in order to perform ^{31}P-NMR measurements. We also performed a cross-contamination experiment, using host tissues and CZ to determine the possible contamination of host tissues by FIZ, and vice versa. For this purpose, we needed uncontaminated samples of both host tissues and zooxanthellae. We used zooxanthellae-depleted host tissues, prepared using bleached *S. pistillata* corals cultured for 6 weeks in the dark. Microscopy observations confirmed that symbiont density was 3 orders of magnitude lower in bleached (6.7 10^3 zoox cm^{-2}) than in normal colonies (2.5 10^6 zoox cm^{-2}). For the

uncontaminated symbionts, we used CZ. For the cross-contamination experiment, we used two identical aliquots of host tissues from bleached *S. pistillata*, and two identical aliquots of CZ. We mixed one aliquot of each, and then separated them again through centrifugation. The two cross-contaminated samples were each compared to the second non-contaminated (control) aliquot. All the procedure was performed at a temperature of 0-4°C, to prevent any metabolic changes involving phosphorus-containing compounds in either of the fractions, such as phosphorus uptake by CZ from the host tissues. Percentage of phosphorus enrichment/loss in the cross-contaminated samples was calculated for two of the main regions of interest presented on Figure 6, phosphate and total phosphonates. Results from the cross-contamination experiment showed that, during centrifugation, zooxanthellae contamination by the host tissue was high, while the host tissues were only slightly contaminated by the zooxanthellae (Fig. 8). Indeed, the two spectra (control and cross-contaminated) were very similar for the host (Fig. 8a), and phosphate and phosphonate contents only increased by 9 and 4%, respectively, due to the cross-contamination. On the contrary, the two spectra of the CZ greatly differed (Fig. 8b), the peaks were shifted, due to a shift in pH (visible on peaks 2 and 3 of PPA on Fig. 8b), and the phosphate content decreased by 39% (from peak 5 to peak 6 on Fig. 8b) while phosphonate content increased by 590%, from 0.07 µmol to 0.48 µmol (Fig. 8c and insert on Fig. 8b). Also, some of the peaks from the animal tissues appeared in the cross contaminated CZ, especially in the phosphonate region (Fig. 8c and insert on Fig. 8b). Also, the peak at -10 ppm, observed for the control CZ, disappeared after cross-contamination (peak 7 on Fig. 8b). Since the cross-contaminated CZ were considered to mimic what happens to FIZ when they are separated from host tissues through centrifugation, we therefore concluded that the separation technique by centrifugation was not suitable for FIZ observations by ^{31}P-NMR, and we then decided to discard the subsequent FIZ spectra. Further confirmation came from the high degree of similarity between the spectra obtained for cross-contaminated CZ and FIZ (Fig. 9). However, our cross-contamination experiment cannot rule out the possibility that phosphonates were

present in FIZ before the separation procedure, perhaps obtained in vivo from host tissue, and further investigations are needed to clarify this.

Figure 6. NMR spectra of 6 known compounds in two solutions, either containing c.a. 5 µmol of aminoethylphosphonic acid (AEP) and phenylphosphonic acid (PPA), or c.a. 5 µmol of PPA, KH_2PO_4, beta-glycerophosphate, adenosine triphosphate (ATP), and polyphosphates. The major peaks were identified from published values and from spectra of each compound alone. The inset on the left shows the AEP peak that was obtained separately, aligned with the rest of the spectra. 1 is the AEP peak, 2 is the PPA peak, 3 is the beta-glycerophosphate peak, 4 is the phosphate peak, 5, 9, and 11 are the polyphosphate peaks, 6 and 7 are the γ-ATP peaks, 8 is the β-ATP peak, and 10 is the α-ATP peak.

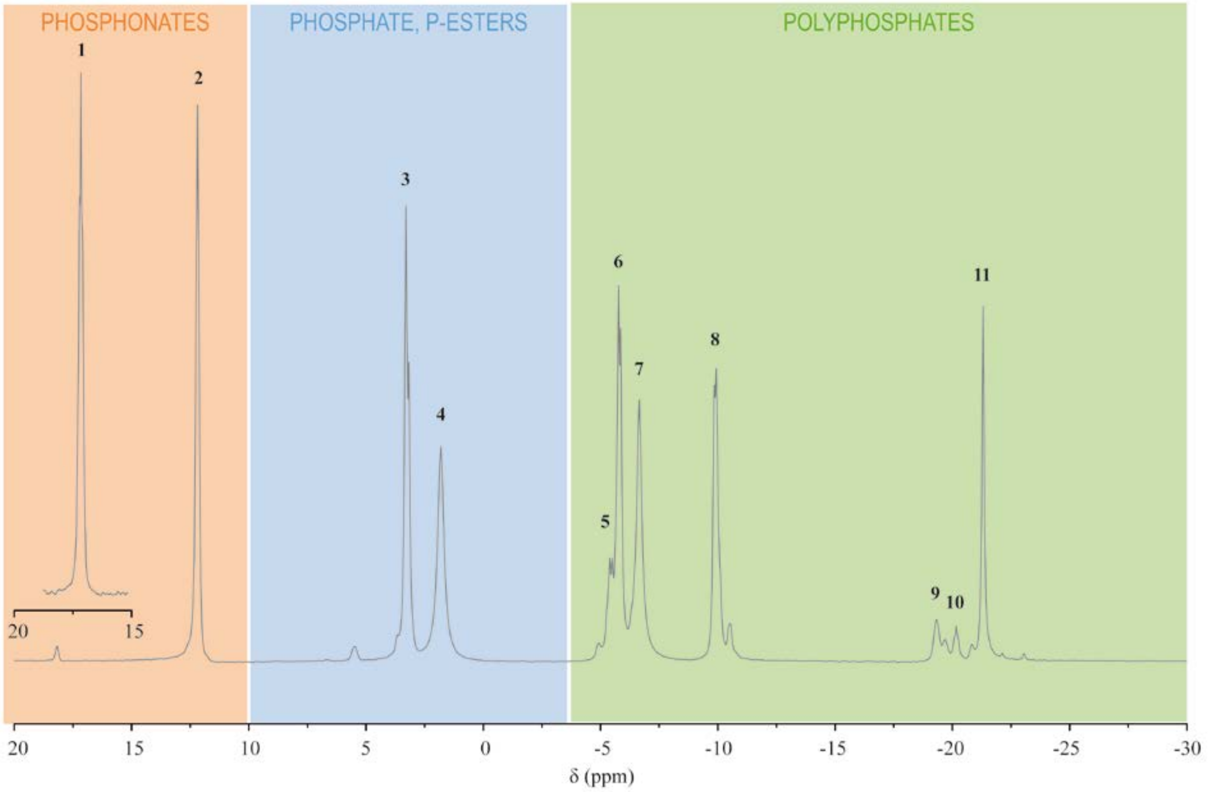

Figure 7. Variation of δ PPA and δ PO_4 with pH.

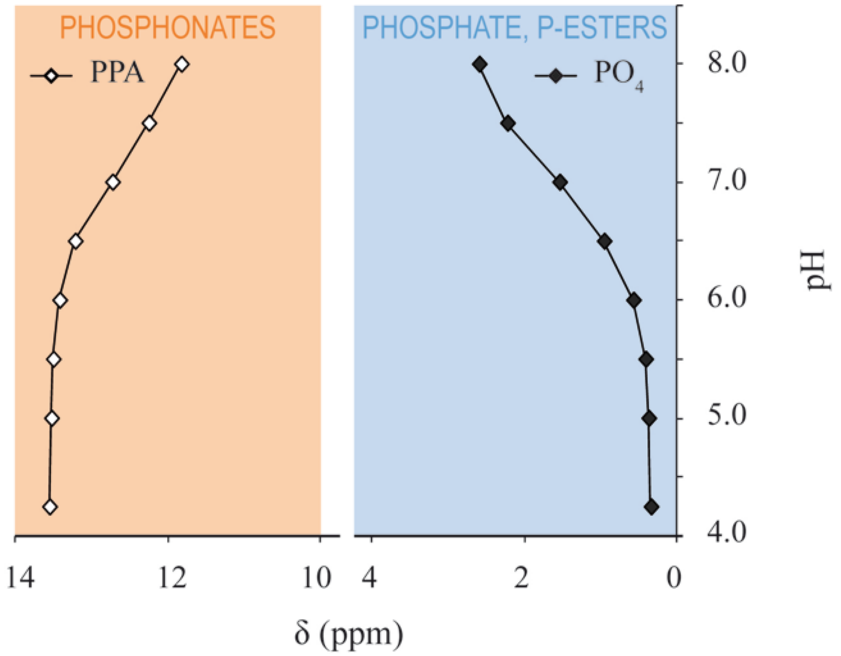

Figure 8. Impact of the centrifugation technique used for the separation of the two components of coral tissues on the ^{31}P-NMR spectra seen via a cross contamination experiment. Extracted aposymbiotic (bleached) *S. pistillata* host tissues were mixed with cultured zooxanthellae (CZ), and then separated through centrifugation. The two cross-contaminated host (**a**) and CZ (**b**) samples (in grey) are each compared to equal quantities of control bleached host tissues and CZ that have not been cross-contaminated (in black). Arrows 1-3: PPA peak (5.0 µmol for CZ, 0.5 µmol for host tissues), arrows 4-6: phosphate peak. The insert on the left-hand side of panel **b** is a close-up of the phosphonate zone of the CZ spectra. Panel **c** is a direct comparison of cross-contaminated host tissues and cross-contaminated CZ. The two spectra were offset for readability.

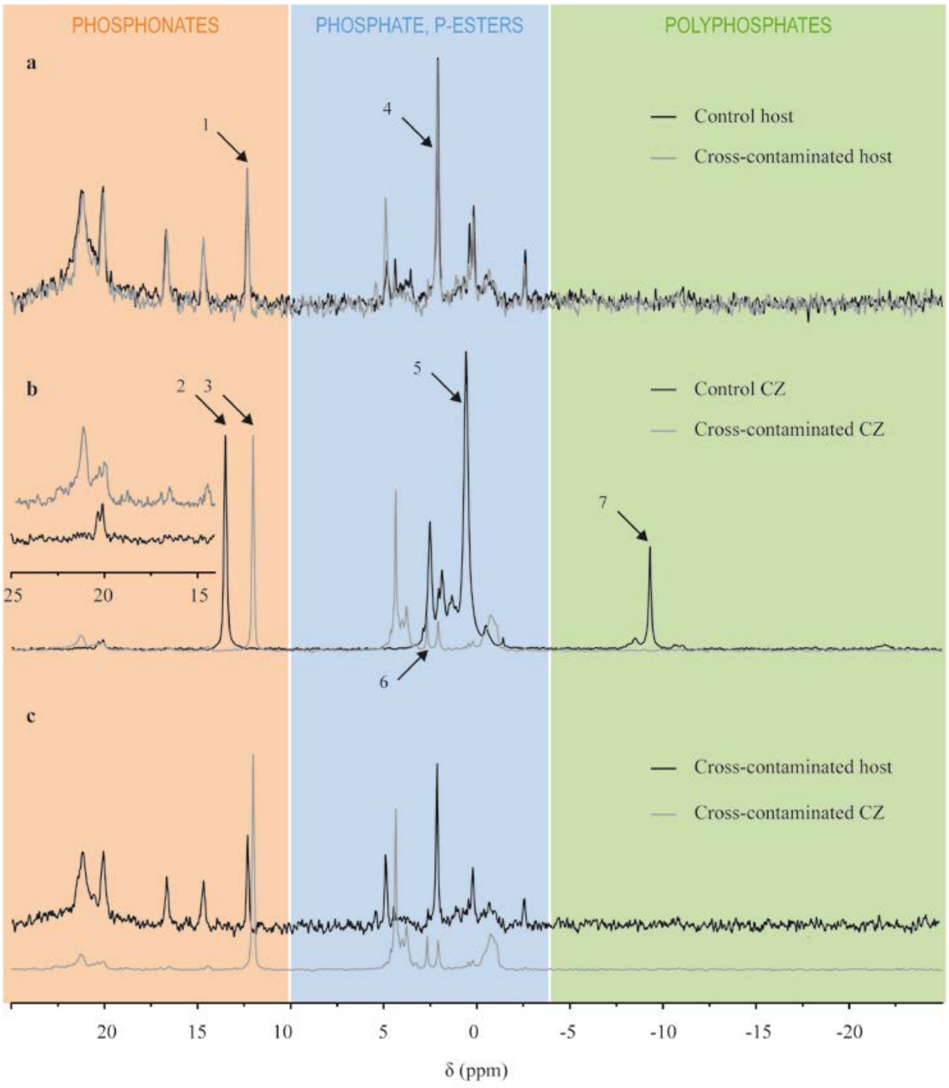

Figure 9. Comparison between NMR spectra of cross-contaminated CZ (in grey) and FIZ (in black). Arrow 1 indicates the PPA standard (5.0 µmol), arrow 2 the phosphate peak, and arrow 3 the 21.2 ppm phosphonate peak.

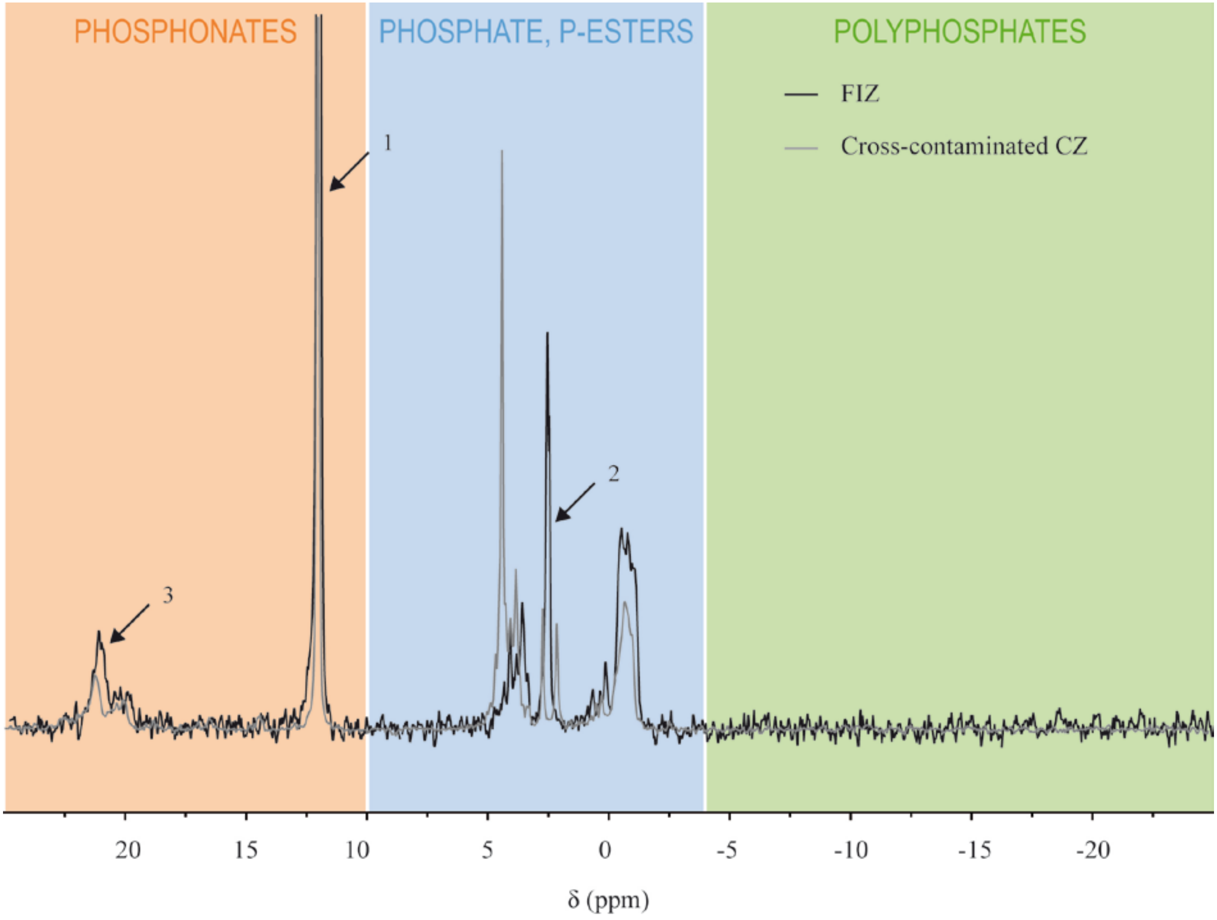

References

Henderson TO, Glonek T, Hilderbrand RL, Myers TC (1972) Phosphorus-31 nuclear magnetic resonance studies of the phosphonate and phosphate composition of the sea anemone, Bunadosoma, sp. Arch Biochem Biophys 149:484-497

Kittredge JS, Roberts E, Simonsen DG (1962) The occurrence of free 2-aminoethylphosphonic acid in the sea anemone, *Anthopleura elegantissima*. Biochemistry 1:624-628

Quin LD (1964) 2-aminoethylphosphonic acid in insoluble protein of the sea anemone *Metridium dianthus*. Science 144:1133-1134

Steen RG (1986) Impact of symbiotic algae on sea anemone metabolism: analysis by in vivo ^{31}P nuclear magnetic resonance spectroscopy. The Journal of Experimental Zoology 240:315-325
